# Health Professional Student Placements and Workforce Location Outcomes: Protocol of an Observational Cohort Study

**DOI:** 10.2196/21832

**Published:** 2021-01-14

**Authors:** Narelle Campbell, Annie Farthing, Susan Witt, Jessie Anderson, Sue Lenthall, Leigh Moore, Chris Rissel

**Affiliations:** 1 Flinders University, Northern Territory Darwin Australia; 2 Flinders University, Northern Territory Alice Springs Australia; 3 Flinders University, Northern Territory Adelaide Australia; 4 Flinders University, Northern Territory Katherine Australia

**Keywords:** remote health, students, training, workforce retention, workforce location, workforce, allied health, allied health professionals, Northern Territory of Australia, rural, nursing, rural employment

## Abstract

**Background:**

The successful recruitment and retention of health professionals to rural and remote areas of Australia is a health policy priority. Nursing or allied health professional students’ learning placements in the Northern Territory (NT) of Australia, most of which is considered remote, may influence rural or remote work location decisions.

**Objective:**

The aim of this study is to determine where allied health professionals and nurses who have had a student placement in the NT of Australia end up practicing.

**Methods:**

This research is an observational cohort study, with data collection occurring at baseline and then repeated annually over 10 years (ie, 2017-2018 to 2029). The baseline data collection includes a demographic profile of allied health and nursing students and their evaluations of their NT placements using a nationally consistent questionnaire (ie, the Student Satisfaction Survey). The Work Location Survey, which will be administered annually, will track work location and the influences on work location decisions.

**Results:**

This study will generate unique data on the remote and rural work locations of nursing and allied health professional students who had a placement in the NT of Australia. It will be able to determine what are the most important characteristics of those who take up remote and rural employment, even if outside of the NT, and to identify barriers to remote employment.

**Conclusions:**

This study will add knowledge to the literature regarding rates of allied health and nursing professionals working in remote or rural settings following remote or rural learning placements. The results will be of interest to government and remote health workforce planners.

**Trial Registration:**

Australian New Zealand Clinical Trials Registry (ANZCTR) ACTRN12620000797976; https://www.anzctr.org.au/ACTRN12620000797976.aspx

**International Registered Report Identifier (IRRID):**

PRR1-10.2196/21832

## Introduction

### Background

Australia’s population predominately lives in its major cities (72%), with another 26% living in regional areas and just 2% living in remote or very remote areas [1]. However, 73% of the Australian landmass is considered *very remote* and a further 13% as *remote* [2], with the *outer regional* and *inner regional* categories respectively covering 11% and 3% of Australia’s land area. The Northern Territory (NT) of Australia is one of the least densely populated areas in the world, with 0.16 people per square kilometer, compared to the overall Australian rate of 3.3 people per square kilometer, 4.1 people per square kilometer in Canada, and 36 people per square kilometer in the United States [3].

The health status of residents in rural and remote Australia is well-known to be worse than that of residents of major cities. Chronic disease and mortality rates are higher and access to health services are poorer [4]. One of the key factors that influences access to health services in rural and remote Australia is the ability to recruit and retain skilled and qualified clinicians [5-8]. There is a maldistribution and shortage of the health workforce, with a lack of health professionals outside major centers being one of the reasons often cited for lower health outcomes of remote and rural Australians. In addition, practicing as a health professional in remote areas is significantly different from practicing in metropolitan centers [9,10]. However, frequently, the curriculum in health professional training programs pays scant attention to preparing students for remote practice [11-13].

The NT has the highest proportion (approximately 30%) of Indigenous residents in Australia, with many living in remote and very remote locations. Aboriginal Australians also carry a higher burden of disease and have greater challenges in accessing health services [14].

The National Strategic Framework for Rural and Remote Health [2] workforce profile demonstrates the difficulties for rural and remote residents in accessing health professionals. For example, it estimates that in rural and remote Australia there are 589 registered nurses per 100,000 population compared to 978 per 100,000 in major cities, and only 64 allied health workers per 100,000 population compared to 354 per 100,000 in major cities [2]. Internationally, the value of the health workforce and the need to significantly increase it has been recognized by the World Health Organization [15].

Undertaking workplace-based learning placements is foundational throughout health professional training programs [16]; in addition, there is a growing body of literature from Australia, Canada, and New Zealand on the value of quality student placements in rural and remote areas and the ways in which placements might positively influence recruitment and retention [17-20]. The successful recruitment and retention of health professionals to rural and remote areas has also become a focus of current health policy. The Australian Commonwealth Government introduced Rural Health Multidisciplinary Training (RHMT) Expansion Program funding in 2016 with a specific focus on nursing and allied health professions. The RHMT Program is designed to encourage the recruitment and retention of rural and remote health professionals by “supporting effective rural training experiences” for health students [21].

Research into the effects of undertaking placements in rural and remote areas and students’ intentions to return to take up positions after graduation has found a positive association between exposure to rural practice and the intention to consider working in a similar situation [7,22-29]. One study also found a positive relationship between rural exposure and return to rural practice, even among those who had taken up positions in a metro area: “there did seem to be a widespread disposition to working in rural areas...even if they were not currently doing so” [30]. Critically, however, there is little known about how intention translates into the uptake of positions in rural and remote places over time.

The career decisions that early-career health professionals make are influenced by a range of factors (eg, [31-34]). Many of these, such as family location and personal career goals, are beyond the control or influence of universities and workplaces. Equally important, the quality of placements in rural and remote areas can be directly influenced by the work of the RHMT Program and the support provided to students, workplaces, and supervisors [29]. The impact of large-scale but locally delivered placement support on long-term remote career location decisions by nursing and allied health professional students has not been investigated.

Internationally, there are relatively few studies on the effects of rural or remote health placements on allied health professionals’ decisions to work in a remote setting. The bulk of the literature regarding work location outcomes focuses on medical students, with most studies reporting that an “organised, well-funded, rural placement or rural clinical school program produced positive associations with increased rural intentions and actual graduate rural employment” [35]. Considerable work also focuses on challenges associated with remote nursing [36].

### Context

Flinders University in Adelaide, Australia, has a long history of delivering education and training to develop the Australian NT health workforce [37,38]. In 2016, Flinders University was awarded a national federal government grant, the RHMT Expansion Program, to provide NT-wide support for work-integrated learning placement students from nursing and allied health professions. The grant encourages rural and remote placements as a strategy for recruitment of health professions to the rural and remote workforce. To evaluate the effectiveness of this program, the planned study will track the career location decisions of former placement students and explore the impact of their NT placements on their work choices.

This research is a 10-year tracking study of the work practice locations of all nursing and allied health students who complete an NT work-integrated learning placement. The study will also investigate the factors that contribute to the work location decisions of the participants and will determine if and how an NT placement influenced career decision making.

### Research Question and Objectives

The overarching research question for this study is as follows: Where do nursing and allied health professionals who have had a student placement in the NT end up practicing? The primary objective of this study is to identify the workplace locations, annually, of nursing and allied health students who completed an NT work-integrated learning placement; this will be conducted for 10 years postgraduation. The secondary objectives of this study are as follows: (1) investigate factors that contribute to work location decisions and (2) determine if and how an NT placement influenced career decisions.

## Methods

### Theoretical Framework and Study Design

The research will use a pragmatist theoretical framework [39-41]. This research is an observational cohort study with data collection occurring at baseline and repeated annually over 10 years, from 2017-2018 to 2029 (see Table 1). The baseline data collection includes a demographic profile of nursing and allied health students and their evaluations of their NT placements (ie, Survey 1: Student Satisfaction Survey). The Student Satisfaction Survey is used nationally by University Departments of Rural Health across Australia [42]. The annual survey (ie, Survey 2: Work Location Survey) has been purpose developed to track work location and the influences on work location decisions.

**Table 1:** Study design showing timing of recruitment and data collection.

Participant cohort	Study activity by year				
	2020	2021	2022	2023-2028	2029
Retrospective recruitment of 2017-2019 students (Cohorts 1-3)	Access to Flinders University Northern Territory (NT) database of completed placements Recruit and obtain consent using last known phone number and email address Survey 1^a^: already undertaken as part of routine quality assurance Survey 2.1^b^	Survey 2.2	Survey 2.3	Survey 2 taken annually up to and including 2026 for Cohort 1 and up to and including 2027 for Cohort 2	Completed
Recruitment of 2020 students (Cohort 4)	Access to Flinders NT database of commencing placements Recruit and obtain consent when sent Survey 1	Survey 2.1	Survey 2.2	Survey 2 taken annually up to and including 2028	Completed
Recruitment of 2021 students (Cohort 5)	N/A^c^	Access to Flinders NT database of commencing placements Recruit and obtain consent when sent Survey 1	Survey 2.1	Survey 2.2	Survey 2 taken annually until 2029 Cohort 5 tracked for 8 years in the workforce
Recruitment of 2022-2027 students (Cohorts 6-11)	N/A	N/A	Access to Flinders NT database of commencing placements Recruit and obtain consent when sent Survey 1	Survey 2.1	Survey 2 taken annually until 2029 Cohort 6 tracked for 7 years in the workforce Cohort 7 tracked for 6 years in the workforce Cohort 8 tracked for 5 years in the workforce Cohort 9 tracked for 4 years in the workforce Cohort 10 tracked for 3 years in the workforce Cohort 11 tracked for 2 years in the workforce
Recruitment of 2028 students (Cohort 12)	N/A	N/A	N/A	Access to Flinders NT database of commencing placements Recruit and obtain consent when sent Survey 1	Survey 2.1

^a^Survey 1 is the Student Satisfaction Survey and is a student evaluation of their placement.

^b^Survey 2 is the Work Location Survey. Note that 2.1 refers to participants’ first year in the workforce or postgraduation, 2.2 is their second year, etc. Participation ends after 10 years in the workforce or postgraduation.

^c^N/A: not applicable; no study activities were performed in this year for these cohorts.

### Participants and Eligibility

Study participants will be students or graduates, over the age of 18 years, of an Australian allied health or nursing training program and will have undertaken an NT placement as a student. Their placement, including any assessment of competence, will be concluded by the time of participation. Individuals who were already employed as a nursing or allied health professional and undertaking a placement as part of a postgraduate qualification will be excluded.

Framed by our pragmatist approach and as part of our due diligence around appropriate research designs, we considered using Australian Health Practitioner Regulation Agency (AHPRA) registration data rather than participant survey data. However, only about half of the professions of interest are registered through the AHPRA: Aboriginal and Torres Strait Islander Health Practitioner, Chinese medicine, chiropractic, dentistry, medical radiation, nursing and midwifery, occupational therapy, optometry, osteopathy, pharmacy, physiotherapy, podiatry, and psychology. Disciplines that are not registered include audiology, dietetics and nutrition, disability, exercise physiology, medical laboratory science, orthotics and prosthetics, paramedicine, social work, and speech pathology. Therefore, relying on AHPRA data to answer our primary question will not provide coverage of all the professions of interest.

The research design relies on access to the contact details of health professional students known to have undertaken work-integrated learning placements in the NT from 2017 onward. Flinders NT holds a secure database with the contact details of the placement students. These details were obtained for the purpose of supporting the placement; however, students consent to the use of their information for educational research purposes, such as our standard quality-improvement Student Satisfaction Survey regarding placement. Access to the database will allow the researchers to recruit participants to the study by contacting current and former students and inviting them to participate in the research.

### Ethics and Trial Registration

Ethics approval was provided by the Flinders University Social and Behavioural Research Ethics Committee (project No. 8245, expiring December 31, 2029). This study was registered with the Australian New Zealand Clinical Trials Registry (ANZCTR) (ACTRN12620000797976).

### Sample Size

In 2017, Flinders NT supported 462 students on placement from more than 20 universities across Australia. The average response rate to the standard quality assurance Student Satisfaction Survey regarding placement is about 30%. Based on 450 students per year undertaking placements and a conservative 15% response rate in the first year from students in the first three cohorts, approximately 203 participants will be added in the first year. Each year, another 450 students will be added to the sampling pool; however, over a 10-year period, we assume that the response rate will decline logarithmically by 1% each year, so that in the tenth year the response rate will be 6%. This equates to a total possible pool of 5400 participants who will each be offered up to 10 surveys, depending on the year they commenced in the study; however, with the declining response rates, we anticipate a final sample of 3175.

### Participant Recruitment

The recruitment process comprises an email invitation to participate. If a message bounces back from an email address, we will contact the participant seeking an email address via the last known telephone number in the database.

The research is comprised of annual online surveys, or phone surveys at the participant’s request. Survey 1 (ie, the Student Satisfaction Survey) is the first survey and is an evaluation of the student placement. The following year, Survey 2 (ie, the Work Location Survey) is sent, which comprises a 10-minute online survey asking about current work location and work location history and includes several questions regarding the influences on work location decisions. All subsequent years will use Survey 2 (ie, the Work Location Survey) to ask about work location and work location history, but this survey will not repeat the initial questions regarding demography and the NT placement.

Participation is voluntary and former students can also opt out of receiving any further surveys. In order to maximize the response rate while avoiding harassment of potential participants, we will send an email invitation to complete the survey, followed by two reminders that also contain the survey link. The reminders will include an option to cease receiving communications from Flinders NT.

We will also advertise the research on our social media sites (ie, Facebook, blog page, and website) in order to maximize exposure of potential participants to the study. This advertising will not contain a link to the survey but will simply describe the study and invite potential former placement students to contact us if they have not received an email invitation to participate.

To summarize, participants will be recruited in one of two ways, depending on their year of NT student placement:

1. Cohorts 1 and 2, which include health professional students known to have undertaken a Flinders NT–supported work-integrated placement during 2017 or 2018, will be contacted via their last recorded email address or phone number and invited to participate.
2. Cohorts 3 to 12, which include health professional students undertaking Flinders NT–supported work-integrated placements from January 2019 and onward, will be emailed an invitation to participate at the conclusion of their placement.

Participants who complete Survey 1 will have their names placed in a random draw to win one of four vouchers valued at Aus $50 (US $38). Participants who complete Survey 2 will have their names placed in a random draw to win one of 10 vouchers valued at Aus $100 (US $76).

### Student Training and Placement

In this study, the intervention is having undertaken a student placement in the NT of at least one week in length. As part of standard accreditation processes, university-level health professional courses are required to demonstrate graduate competence in the application of knowledge and skills to patients and consumers of health care. Workplace-based placements, sometimes known as work-integrated learning activities, are a cornerstone of the curriculum because the students are working in health care workplaces under the supervision of a qualified health professional, learning to deliver the graduate competencies of the profession [43,44].

The supervision requirements, length, curriculum, and assessment of student placements vary according to the needs of the specific profession [12]. Whether the university pays the workplace or the workplace supervisor for providing the placement also varies. We recognize that the intervention includes student placement experiences that are not homogenous. From the perspective of workplaces, it is commonly assumed that student placements are a mutually beneficial recruitment strategy, allowing both the workplace and the student to consider the suitability and attractiveness of employment once they graduate [23,24,27,42,45].

### Retention Strategies

To maximize participation in the study, we will use a set of effective cohort retention strategies tested in previous cohort studies (eg, the Communicating Healthy Beginnings Advice by Telephone [CHAT] trial [46]). For example, we will send a thank-you e-card and birthday card to all participants. The relationship of the student with the placement coordinator or workplace supervisor may also influence survey participation, and we will seek ways to incorporate individual anecdotes into communications with students.

### Outcomes

The primary outcome of the study is the workplace location, assessed using the Modified Monash Model (MMM) [47], with MMM 4 and 5 considered rural and MMM 6 and 7 considered remote. The MMM defines whether a location is urban, rural, remote, or very remote, based on the Australian Statistical Geography Standard—Remoteness Areas (ASGS-RA) framework. The model measures remoteness and population size on an MMM category scale, ranging from MMM 1 to MMM 7. MMM 1 is a major city and MMM 7 is very remote. Any health service provision in areas classified as MMM 4 to 7, whether full time or part time, in the previous 12 months will be considered a rural or remote workplace location.

Baseline data (ie, Survey 1: Student Satisfaction Survey) will be collected following student placements and will include age, gender, degree program in which student is enrolled, length of placement, placement location, Indigenous status, NT residency, and rural origin.

### Data Analysis

To clarify the representativeness of the sample participants who respond each year, we will compare the distribution of the responders with the nonresponders in terms of age, gender, profession, length of placement, and Indigenous status. Descriptive analysis of deidentified aggregated data is planned. This will include survey response rates; number of student placements by profession and location, using the MMM and not town location; length and number of placements; gender; rural origin; NT residency; and influences on placement. Changes in work location will be analyzed by location. Factors associated with remote work location (ie, MMM 4 to 7 versus MMM 1 to 3) will be analyzed using logistic regression.

## Results

This study will generate unique data on the remote and rural work locations of nursing and allied health professional students who will have had placements in the NT of Australia. It will be able to determine what are the most important characteristics of those who take up remote and rural employment, even if outside the NT, and identify barriers to remote employment.

## Discussion

The question of how many allied health and nursing students, as well as their characteristics, who have had a remote student placement go on to work remotely is a gap in the literature on remote work locations, which more typically focuses on medical students and to a lesser extent on nursing students. Filling this gap is important, given the federal funding invested in the RHMT Expansion Program as a strategy to grow a local remote health workforce. Given the national policy priority to recruit and retain health professionals in remote and rural areas, new insights into strategies to achieve this are needed. It is recognized internationally that building a remote and rural health workforce is a common challenge [48].

The analyses will be able to determine what are the most important characteristics of those who take up remote and rural employment, even if outside the NT, while adjusting for potential confounders, and will identify independent barriers to remote employment. A meta-synthesis of recruitment and retention of occupational therapists and physiotherapists in rural areas found that the availability of, and access to, practice supports; opportunities for professional growth; and an understanding of the context of rural practice were important [20]. Further, the calculation of the proportion of students who have had a placement in a remote setting and went on to work remotely is important for workforce planning. One study reported that 1 year after graduation, half of the allied health students who had had a rural placement were working in a rural or remote location, compared to 23.7% of all graduates from these disciplines [49]. It remains to be determined what proportion of students undertaking a remote placement will be working in a remote location in the short, medium, or long term after graduation.

The very low population density in the NT makes it a unique context to study remote health workforce issues. How the factors influencing the selection of remote health workplace locations compared to rural settings needs further study.

Possible limitations of this study revolve around potential low participation rates, which could bias outcomes. Prompts, reminders, and acknowledgements (eg, birthday cards) will assist, but it may be that those most interested in rural and remote work will respond and that those who either had a negative experience of remote work while on placement or in some other context will choose not to participate. General information provided to the wider pool of nursing and allied health professional students about the study and the importance and benefits of rural and remote work will encourage participation in the study and will, more generally, promote interest in placements in the NT.

## Abbreviations

AHPRA: Australian Health Practitioner Regulation Agency
ANZCTR: Australian New Zealand Clinical Trials Registry
ASGS-RA: Australian Statistical Geography Standard—Remoteness Areas
CHAT: Communicating Healthy Beginnings Advice by Telephone
MMM: Modified Monash Model
NT: Northern Territory
RHMT: Rural Health Multidisciplinary Training
